# Supplementary material for: Daily Adequate Intake of Fruit and Vegetables and All-Cause, Cardiovascular Disease, and Cancer Mortalities in Malaysian Population: A Retrospective Cohort Study
Source: Nutrients. 2024 Sep 21;16(18):3200. doi: 10.3390/nu16183200 (PMC11435129; doi:10.3390/nu16183200)
Supplement: Supplementary file 1 [file nutrients-16-03200-s001.zip › nutrients-3182216-supplementary.pdf]

Supplementary Table S1. The associations between fruit intake and vegetable intake with all-cause mortality, CVD mortality and cancer mortality among the NHMS 2011 respondents

	**All-cause Mortality** (N=18,211, N^O=1,809)	**All-cause Mortality*** (N=19,903, N^O=1,501)	**CVD Mortality** (N=18,211, N^O=374)	**CVD Mortality *** (N=17,903, N^O=312)	**Cancer Mortality** (N=18,211, N^O=216)	**Cancer Mortality *** (N=17,903, N^O=178)
**Vegetable Intake**						
Unadjusted HR (95% CI)	0.98 (0.82 – 1.19)	1.02 (0.82 – 1.26)	0.87 (0.58 – 1.31)	0.89 (0.56 – 1.44)	1.27 (0.81 – 1.98)	1.13 (0.65 -1.97)
Multiple Cox Regression adjusted HR (95% CI)[a]	1.19 (0.88 – 1.61)	1.25 (0.88 – 1.76)	0.81 (0.40 – 1.63)	0.81 (0.36 – 1.80)	0.98 (0.41 – 2.34)	0.93 (0.34 – 2.55)
Multiple Cox Regression adjusted HR (95% CI)[b]	1.21 (0.96-1.54)	1.26 (0.97-1.64)	0.99 (0.59 – 1.68)	0.99 (0.55 – 1.78)	0.79 (0.47 – 1.31)	0.94 (0.43 – 2.03)
**Fruit Intake**						
Unadjusted HR (95% CI)	0.92 (0.77 – 1.10)	0.82 (0.67 – 1.00)	1.07 (0.70 – 1.62)	1.14 (0.71 – 1.82)	1.32 (0.89 – 1.96)	1.04 (0.63 – 1.71)
Multiple Cox Regression adjusted HR (95% CI)[c]	0.84 (0.68 – 1.03)	0.76 (0.61 – 0.95)	0.93 (0.57 – 1.53)	0.91 (0.52 – 1.58)	0.90 (0.59 – 1.37)	0.81 (0.50 – 1.33)
Multiple Cox Regression adjusted HR (95% CI)[b]	0.87 (0.69-1.09)	0.80 (0.62-1.03)	1.00 (0.59 – 1.70)	0.99 (0.54 – 1.80)	0.92 (0.49 – 1.91)	0.69 (0.38 – 1.26)

CVD= cardiovascular disease; NHMS = National Health and Morbidity Survey; N= number of participants; N^O= number of death; *=excluding mortality in the first 2 years; HR = hazard ratio; 95% CI = 95% confidence interval

a = adjusted for sex, ethnicity, age, residential area, marital status, education level, monthly household income, obesity, alcohol intake, smoking, physical activity, diabetes, hypertension, hypercholesterolemia, daily adequate fruit intake

[b] = adjusted for sex, ethnicity, age, residential area, marital status, education level, monthly household income, obesity, alcohol intake, smoking, physical activity, diabetes, hypertension, hypercholesterolemia, and daily adequate fruit intake x daily adequate fruit intake

[c] = adjusted for sex, ethnicity, age, residential area, marital status, education level, monthly household income, obesity, alcohol intake, smoking, physical activity, diabetes, hypertension, hypercholesterolemia, daily adequate vegetable intake
